# Congenital erythropoietic porphyria presenting with recurrent epistaxis: a case report

**DOI:** 10.1186/s13256-023-04204-5

**Published:** 2023-11-14

**Authors:** Javeriah Khan, Muhammad Usman Hashmi, Nabeeha Noor, Ahmad Jamal Khan, Oadi N. Shrateh, Muhammad Junaid Tahir

**Affiliations:** 1 Pakistan Institute of Medical Sciences, Islamabad, Pakistan; 2 Rawalpindi Medical University, Rawalpindi, Pakistan; 3 Sahiwal Medical College, Sahiwal, Pakistan; 4 Lahore General Hospital, Lahore, Pakistan; 5 Faculty of Medicine, Al-Quds University, Jerusalem, Palestine; 6 Shaukat Khanum Memorial Cancer Hospital and Research Centre, Lahore, Pakistan

**Keywords:** Gunther disease, Epistaxis, Thrombocytopenia, Enzyme mutation, Case report

## Abstract

**Background:**

Congenital erythropoietic porphyria (CEP), also known as pink tooth or Gunther disease, is a rare hereditary disorder caused by an enzyme mutation in the heme biosynthesis pathway, which leads to the accumulation of immature and non-physiological protoporphyrin rings in various tissues. CEP is characterized by sun-exposed bullous skin lesions, hemolytic anemia, red/brown urine, and teeth staining.

**Case presentation:**

We present a unique case of a 10-year-old Asian boy with CEP who presented with recurrent epistaxis, an unusual presentation for this condition. Based on clinical presentation and laboratory findings, including elevated urine uroporphyrin and coproporphyrin I and III levels, microcytic anemia, a higher red cell distribution width (RDW), and a lower platelet count, a thorough assessment and detailed workup resulted in a diagnosis of CEP. The patient underwent a successful splenectomy and recovered without any complications.

**Conclusion:**

This case report aims to raise awareness among healthcare professionals about the uncommon and atypical presentation of CEP and its management options.

## Background

Congenital erythropoietic porphyria (CEP), also known as pink tooth or Gunther disease, is a rare hereditary disorder. So far, only a few hundred cases have been documented in the literature [1]. It is an autosomal recessive disease caused by an enzyme mutation, i.e., uroporphyrinogen III synthase (UROS) in the heme biosynthesis pathway, ferrochelatase, another enzyme incorporating iron into the protoporphyrin ring, is also affected [2]. Therefore, immature and non-physiologic protoporphyrin rings are formed and ultimately accumulate in different tissues, including skin, bone marrow, and blood, and cause various symptoms of porphyria [3].

Generally, the onset is between the neonatal period and the first 10 years, and photosensitivity is the most prominent symptom, which usually surfaces in adolescence, along with other hematological complications such as neonatal jaundice, transfusion-dependent hemolytic anemia, thrombocytopenia, and splenomegaly. However, congenital erythropoietic porphyria does not typically include neurovisceral symptoms such as abdominal pain, psychiatric manifestations, seizures, or neurologic symptoms [4].

We present a unique case of CEP in a 10-year-old boy who presented with recurrent epistaxis for the last 6 months. After a thorough assessment and detailed workup, he was diagnosed with congenital erythropoietic porphyria. This patient's presentation was unusual and atypical, and the patient was prone to developing anesthesia-related complications due to structural deformities of the body. Therefore, the authors report this case to raise awareness among fellow healthcare professionals. We hope our findings can advance knowledge about congenital erythropoietic porphyria, its rare and atypical presentation, and treatment options.

## Case presentation

Our patient is a 10-year-old male of Asian descent who presented to the outpatient department of the hospital with an episode of epistaxis 1.5 months back. He has had multiple episodes of epistaxis for the last six months. The patient also suffered from skin blister formation which was exacerbated by exposure to sunlight.

The patient was born into a nonconsanguineous marriage. None of his 2 siblings were affected. Both parents were too phenotypically normal.

His gestational period and delivery remained uneventful. The patient weighed 2.5 kg at birth and had a head circumference of 38 cm (or 14.9 in.). At delivery, the baby's APGAR score was 7, and his height percentile was in the 50th percentile, but his weight percentile was in the 40th percentile. He made normal progress and achieved the age-appropriate developmental milestones. However, he did not receive any vaccination except the BCG vaccine.

A detailed history revealed that the child had multiple visits to the local doctor for skin blisters on his hands, feet, face, and neck and a change in the hue of his urine. However, he was not evaluated thoroughly and did not receive any treatment other than advice to avoid sun exposure and application of moisturizing creams. Other than this patient did not have any chronic medical problems or any surgery.

The patient was of Asian descent. He was born in Gilgit Baltistan, an area in the North of Pakistan. The patient did not have any problems throughout his childhood except recurrent skin blisters formation. He enjoyed playing outside in nature but then he was told not to play outside due to photodermatitis. He is in his primary school and enjoys making hand-made wild grass baskets. He lives with his family and has a poor socio-economic status. He eats a traditional diet but mostly fresh fruits and nuts. He has a normal sleep and is not sexually active. He has no history of alcohol use, tobacco smoking, or other recreational substance use.

**Fig. 1:**
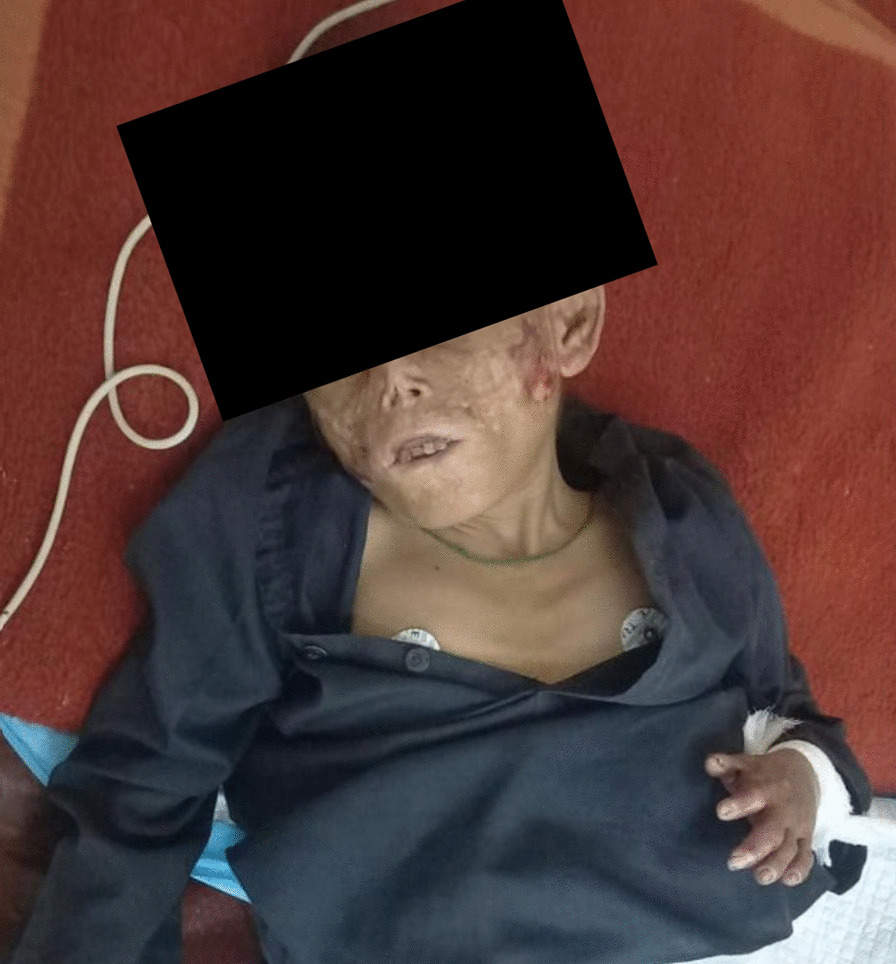
Thickened skin with hypopigmentation and hyperpigmentation, as well as scarring over sun-exposed areas, face hypertrichosis, and discoloration of the teeth

The complete general physical examination showed that the patient was well-oriented. When the patient arrived, his vital signs were normal. The heart rate was 90 beats per minute, the blood pressure was 90/65 mmHg, the body temperature was 98 degrees Fahrenheit, and the breathing rate was 18 breaths per minute. However, he had pallor, brown teeth, obvious facial scars, a distorted visage, and thin, sparse hair, as shown in Fig. 1.

**Fig. 2:**
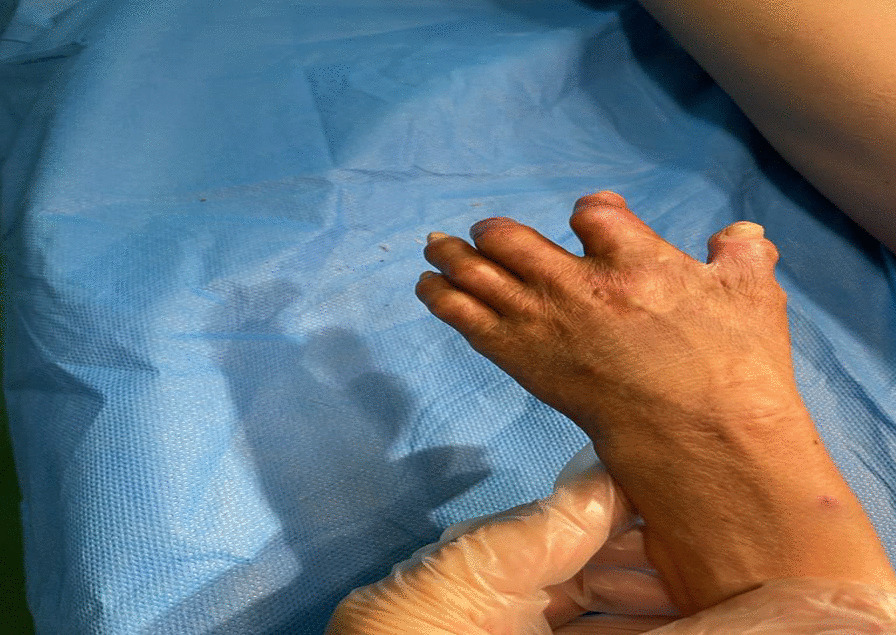
Severe finger mutilation with scarring and hypertrichosis on sun-exposed parts

Additionally, his right index finger had anonychia, onycholysis, and brachydactyly, as illustrated in Fig. 2. The child's head circumference measured 115 cm, weight was 20 kg (falling within the 5th percentile for weight), and his height stood at 120 cm (in the 10th percentile for height).

He had a soft, non-tender abdomen with a palpable spleen and liver. The full neurological testing yielded completely normal results. The evaluation included cranial nerve evaluation, motor function, sensory function, cerebellar function, and psychological well-being. Notably, no unusual findings were identified in any of these domains. The rest of the review of the review of the systems was also unremarkable.

Based on the observations of history and physical examination, a presumptive diagnosis of chronic blistering cutaneous porphyria was established. So, further laboratory investigations were performed to confirm the underlying etiology. His total urine uroporphyrin and coproporphyrin I and III levels were found to be significantly elevated (Table 1).

**Table 1:** Porphyrins profile in 24-h urine and blood

Test	Level	Reference range	Unit
Uroporphyrins	8720	Up to 30	Nmol/24 h
Coproporphyrin-I	2611	Up to 168	Nmol/24 h
Heptacarboxyporphyrin	182	Up to 9	Nmol/24 h
Hexacarboxyporphyrin	105	Up to 8	Nmol/24 h
Pentacarboxyporphyrin	455	Up to	Nmol/24 h
Erythrocyte porphyrin	790	Up to 70	Microgram/dL
Uroporphyrinogen III synthase (URO-synthase)	Undetectable		

*Nmol* nanomoles, *dL* deciliter

Moreover, he also had microcytic anemia, a higher red cell distribution width (RDW), and a lower platelet count. The peripheral blood film revealed microcytic, hypochromic red blood cells (RBCs) with anisocytosis, poikilocytosis, polychromasia, and basophilic stippling. Additionally, reticulocytosis, absence of haptoglobin, increased unconjugated bilirubin, and increased fecal urobilinogen were detected. The laboratory investigations on admission are summarized in Table 2.

**Table 2:** Summary of laboratory investigations on admission

Test	Result	Units	Reference ranges
TLC	2.29	× 10^9^/L	4–11
Red cell count	3.12	Million/micro	4–5.2
Hemoglobin	4.1	g/dL	11.5–15.5
Platelet count	44	× 1000/micro	170–450
RDW-CV	22.8	%	10–15
Mean platelet volume	5	Femto liter	8.9–11.8
Reticulocytes	3	%	0.5–2
Serum iron	228	Ng/mL	7–140
Alkaline phosphatase	206	U/L	Up to 135
ALT	96.4	U/L	Up to 42
Indirect bilirubin	5.1	mg/dL	0.2–0.8
CRP	49	mg/dL	Up to 5.0 mg/L

*TLC* total leukocyte count, *RDW-CV* red cell distribution width—coefficient of variation, *ALT* alanine transaminase, *CRP* C-reactive protein, *U/L* units per litter, *mg/dL* milligrams per deciliter, *Ng/mL* nanograms per milliliter, *g/dL* grams per deciliter

Consequently, the diagnosis of CEP was confirmed by the presence of characteristic clinical features, raised porphyrins in blood, and urine with a predominance of uroporphyrin I and coproporphyrin I. Genetic testing was not performed due to unavailability.

The epistaxis was considered a consequence of thrombocytopenia. Hence, he was advised to continue taking the medications as prescribed and use protective clothing and sunscreen when exposed to direct sunlight. However, his condition did not improve. Therefore, it was planned to perform splenectomy to prevent the breakdown of platelets and other blood cells by the spleen. Hence, surgery was performed after a comprehensive pre-operative workup and optimization of the patient. The surgery went uneventfully, and the patient experienced a smooth recovery period.

The dosage of the medications administered during the period of hospital stay was as follows: nalbuphine (3 mg), midazolam (1 mg), dexamethasone (2 mg), and ceftriaxone (150 mg). The patient received medication via the intravenous (IV) route throughout their 5-day hospital stay. Additionally, the patient received transfusions, including 2 units of Platelet mega-kit, 2 units of red cell concentrate (RCC), and 1 unit of fresh frozen plasma (FFP).

He was discharged on the 5th postoperative day, with the instructions to follow up after one week. During all his subsequent follow-up visits, he was monitored by thorough clinical evaluation, complete blood count, and serial measurements of porphyrin levels in plasma, erythrocytes, and urine. The parents were advised to protect their children's skin and eyes from the sun and minor trauma. The patient did not report epistaxis, and the follow-up laboratory investigations also revealed the resolution of thrombocytopenia with a platelet count of 188 per microliter. In the subsequent follow-up visits, we did not observe any novel complications.

Following a successful splenectomy and early post-operative recovery, the patient was closely monitored for more than 6 months to assess treatment results and the long-term stability of his condition. During this time, the patient's general health and quality of life improved significantly. Notably, he reported no further development of epistaxis, and the skin blistering, which had been worsened by sunlight exposure. Laboratory findings showed a gradual normalization of hemoglobin levels, red cell distribution width (RDW), and platelet counts, indicating that the patient's anemia improved.

## Discussion

Congenital erythropoietic porphyria (CEP) is a rare genetic disorder that often goes undiagnosed due to its variable clinical presentation [1]. In this case report, we provide a convincing example of congenital erythropoietic porphyria (CEP) in a 10-year-old juvenile boy who presented in an uncommon and atypical manner, with recurrent epistaxis as a prominent symptom. While CEP is an uncommon genetic condition caused by a lack of uroporphyrinogen III synthase, resulting in the buildup of aberrant protoporphyrin rings, this case stands out owing to its unique clinical presentation. CEP is typically characterized by sun-induced skin lesions, hemolytic anemia, colored urine, and tooth discoloration. However, our patient's repeated epistaxis is a rare and unreported clinical manifestation of this illness. This study highlights the value of considering CEP as a differential diagnosis, especially in instances with unusual symptoms, and highlights the diagnostic difficulties. Furthermore, the successful splenectomy that was performed to address this patient's disease points to a prospective course of action in this condition.

According to a study by Katugampola et al., the time from the first symptom to diagnosis can range from eight days to 48 years, with an average delay of 8.1 years [4]. This delay is similar to the case of a 10-year-old patient described in the present study, who experienced symptoms at age two but was not properly diagnosed due to a primary care physician's lack of experience and awareness of this rare disorder. Therefore, CEP requires careful consideration in cases of atypical presentations to prevent misdiagnosis and delayed treatment. The diagnosis of CEP involves clinical and laboratory evaluation to identify cutaneous symptoms, hemolytic anemia, and excess porphyrins in erythrocytes, plasma, and urine, with an increase in fecal porphyrins [5]. The presence of familial pathogenic UROS gene variants is indicated to confirm the diagnosis of CEP [6]. In the case presented, there was an increase in urine and erythrocyte uroporphyrin I and coproporphyrin I, consistent with the previous case report [7]. Skin and bone marrow biopsies are not required for diagnosis as they do not yield specific findings [8]. We acknowledge a limitation in our case report: the lack of genetic testing to validate the precise pathogenic mutations linked with Congenital Erythropoietic Porphyria (CEP). The lack of genetic testing at our local hospital posed a barrier to determining the underlying genetic alterations responsible for this patient's ailment. By finding mutations in genes such as uroporphyrinogen III synthase (UROS), genetic testing can help confirm CEP.

CEP can be difficult to differentiate from other porphyrias and skin disorders, such as hepatoerythropoietic porphyria, porphyria cutanea tarda, variegate porphyria, hereditary coproporphyria, erythropoietic protoporphyria, X-linked protoporphyria, lead poisoning, and bullous skin disorders. Despite overlapping symptoms, there are distinct differences that aid in the diagnosis of CEP [9].

The symptoms of hepatoerythropoietic porphyria (HEP) are similar to those of CEP. They can include skin blistering, photosensitivity, and hypertrichosis (excessive hair growth), but HEP is typically milder and may present later in life. In contrast, CEP is typically severe and presents early in life. Additionally, a predominantly raised heptacarboxyporphyrin, hexacarboxyporphyrin, pentacarboxyporphyrin in urine and plasma, and a uroporphyrinogen decarboxylase (UROD) mutation helps to distinguish HEP from CEP [8].

Porphyria cutanea tarda (PCT) is the most common type of porphyria and is characterized by skin blistering and photosensitivity, similar to CEP. However, the erythrocyte porphyrin level distinguishes PCT from CEP biochemically. The erythrocyte porphyrins are markedly elevated in CEP but typically normal or modestly elevated in PCT. PCT is also associated with other underlying conditions such as hepatitis C virus infection, alcoholism, and hemochromatosis [8].

Variegate porphyria (VP) can be distinguished from CEP by normal or only modestly elevated erythrocyte porphyrins. In contrast, these are significantly high in CEP. Hereditary coproporphyria (HCP) usually presents with neurovisceral manifestations and, less commonly, with blistering skin lesions. Conversely, CEP usually presents with skin lesions in almost all cases. In HCP, plasma porphyrin levels are typically normal but are anticipated to be elevated in the presence of skin lesions [8]. Furthermore, erythrocyte porphyrin levels are normal or only mildly elevated.

Patients with CEP have an increased risk of developing anesthesia-related complications including drug-induced photosensitivity, the risk of acute attacks, and difficulties in airway management. Patients with CEP are highly sensitive to light, which can lead to blistering and scarring of the skin. Certain drugs used during anesthesia, such as barbiturates, can exacerbate this photosensitivity. In addition, CEP patients have an increased risk of developing acute porphyric attacks during anesthesia, which can result in life-threatening complications. These attacks are triggered by various factors, including certain medications, fasting, and stress. Additionally, the development of phototoxicity has been linked to operating room lights that emit blue wavelengths of ultraviolet light. The acrylate filters can be used, along with postoperative recovery in a dimly lit area, to protect the patient from phototoxic damage.

Airway management can also be challenging in CEP patients due to facial and airway edema. This is especially true for patients who have a history of previous acute attacks or chronic facial edema. Therefore, a careful airway assessment should be conducted before anesthesia to avoid complications. A C-Mac video laryngoscope and flexible fiberoptic bronchoscope can be used for endotracheal intubation, and a sealing facemask should be used to ventilate patients [9].

A variety of substances and factors can trigger an acute porphyric crisis. Fasting, dehydration, infections, psychological stress, physiologic hormone variations, excessive alcohol consumption, and particular drugs are a few of these. Counseling before surgery can help reduce physiological stress and anxiety. To shorten the length of the fast, the patients ought to come first or second on the operating room list [9].

Currently, the only curative treatment option is allogeneic bone marrow transplantation. In patients who do not undergo this procedure, other options may include avoidance of sunlight exposure by opting for nighttime jobs, use of sunscreens containing zinc oxide or titanium oxide, vitamin D supplementation, and use of sunglasses to avoid ocular complications. Pharmacologic photoprotection has only been tested in Erythropoietic protoporphyria (EPP) so far hence we cannot comment on its efficacy for CEP [5]. In some patients with hypersplenism and excessive circulating RBC trapping and destruction, splenectomy may reduce the need for transfusions. Additionally, thrombocytopenia brought on by hypersplenism may be successfully treated with a splenectomy [8]. Hence, we planned a splenectomy for this patient, and his symptoms caused by anemia and thrombocytopenia were alleviated after the splenectomy.

It is also important to note that CEP is a lifelong condition, and patients will require regular monitoring and treatment throughout their lifetime. Healthcare professionals can provide better patient care and improve their quality of life by recognizing the disease at its earliest stages, educating on preventive measures, and providing psychosocial support.

## Conclusion

Early detection of CEP symptoms is crucial for accurate diagnosis and treatment. While CEP symptoms may overlap with other porphyrias, specific characteristics such as age of onset, severity, laboratory test patterns, gene mutations, and enzyme abnormalities can aid in diagnosis. A complete evaluation by a healthcare professional, including a detailed medical history, physical examination, and laboratory tests, is necessary for an accurate diagnosis. Due to drug-induced photosensitivity, acute attack risk, and airway management challenges, anesthesia in CEP patients can be difficult and requires careful planning to avoid complications.

## Data Availability

The data used to support the findings of this study are available from the corresponding author upon reasonable request.

## Abbreviations

CEP: Congenital erythropoietic porphyria
UROS: Uroporphyrinogen III synthase
HEP: Hepatoerythropoietic porphyria
UROD: Uroporphyrinogen decarboxylase
PCT: Porphyria cutanea tarda
VP: Variegate porphyria
EPP: Erythropoietic protoporphyria
RBC: Red blood cell
